# Formação em pesquisa qualitativa nos programas de pós-graduação em Saúde Coletiva: panorama no Brasil

**DOI:** 10.1590/0102-311XPT116723

**Published:** 2024-09-16

**Authors:** Dais Gonçalves Rocha, Nilza Rogéria Andrade Nunes, Giannina do Espírito-Santo, Maria Lúcia Magalhães Bosi

**Affiliations:** 1 Universidade de Brasília, Brasília, Brasil.; 2 Faculdade de Medicina, Universidade Federal do Ceará, Fortaleza, Brasil.; 3 Pontifícia Universidade Católica do Rio de Janeiro, Rio de Janeiro, Brasil.; 4 Comissão Nacional de Ética em Pesquisa com Seres Humanos, Águas Claras, Brasil.

**Keywords:** Pesquisa Qualitativa, Educação de Pós-graduação, Saúde Coletiva, Qualitative Research, Graduate Education, Public Health, Investigación Cualitativa, Educación de Postgrado, Salud Colectiva

## Abstract

Este artigo analisa as evidências sobre a formação em pesquisa qualitativa dos programas de pós-graduação em Saúde Coletiva (PPGSC) credenciados pela Coordenação de Aperfeiçoamento de Pessoal de Nível Superior (CAPES), no Brasil, no ano de 2021. Para realizar o mapeamento das disciplinas foi consultada a Plataforma Sucupira para a localização dos PPGSC e busca nos portais institucionais. Foram identificadas 98 disciplinas e utilizando a análise temática reflexiva foi possível agrupar a produção dos dados e caracterização das disciplinas nas seguintes macrocategorias de análise: (1) carga horária e modalidade de oferta (obrigatória ou eletiva); (2) objetivos de ensino-aprendizagens; (3) conteúdos (com destaque para investigação dos paradigmas científicos ou diferentes abordagens teórico-metodológicas da investigação qualitativa); e (4) metodologias de ensino-aprendizagens. Dos resultados, destacam-se que: apenas 40,4% são obrigatórias e predominaram os objetivos de ensino-aprendizagem com foco na instrumentalização e habilitar os(as) pós-graduandos (as) na construção dos projetos de pesquisa. 59,3% não informaram o tipo de metodologia de ensino-aprendizagem. Há uma lacuna quanto à utilização das tecnologias e ambientes digitais e predomínio do formato textual. Constatou-se fragilidade na formação epistemológico-teórica. A aprendizagem do paradigma positivista predomina em relação ao paradigma interpretativo de teorias/tradições críticas. Ao final, são sistematizados elementos para um itinerário formativo com graus crescentes de complexidade, intencionalmente estruturado e desenvolvido em um ambiente institucional que propicie decolonialidade e reparação epistêmica.

## Abstract (en)

This article analyzes the evidence of qualitative research training in graduate programs in Public Health accredited by Brazilian Coordination for the Improvement of Higher Education Personnel (CAPES) in Brazil in 2021. To map out the disciplines, the Sucupira Platform was accessed to locate the graduate programs in Public Health, and the institutional portals were searched. A total of 98 courses were identified. Using reflective thematic analysis, it was possible to group the data production and the characterization of the courses into the following macro categories of analysis: (a) workload and type of course offered (mandatory or optional); (b) teaching-learning objectives; (c) content (with emphasis on the investigation of scientific paradigms or different theoretical-methodological approaches to qualitative research); (d) teaching-learning methodologies. The results show that only 40.4% of the courses are mandatory and the predominant teaching-learning objectives focus on instrumentalization and enabling graduate students to create research projects. In total, 59.3% of the courses did not inform the type of teaching-learning methodology. There is a gap in the use of digital technologies and spaces and a predominance of the text-based approach. Furthermore, the epistemological-theoretical training is fragile. The learning of the Positivist paradigm predominates over the interpretive paradigm of critical theories/traditions. The article concludes by systematizing elements for a training itinerary with increasing degrees of complexity, intentionally structured and developed in an institutional environment that fosters decoloniality and epistemic reparation.

## Resumen

En este artículo se analiza la evidencia sobre la formación en investigación cualitativa de los programas de posgrado en Salud Colectiva acreditados por la Coordinación de Perfeccionamiento de Personal de Nivel Superior (CAPES) en Brasil, en 2021. Para mapear las disciplinas, se consultó a la Plataforma Sucupira con el intento de encontrar los programas de posgrado en Salud Colectiva y buscar las páginas institucionales. Se identificaron 98 disciplinas, y a partir del análisis temático reflexivo, la producción de datos y la caracterización de las disciplinas se agruparon en las siguientes macro categorías de análisis: (a) carga de trabajo y modalidad de oferta (obligatoria o electiva); (b) objetivos de enseñanza-aprendizaje; (c) contenidos (con énfasis en la investigación de paradigmas científicos o diferentes enfoques teórico-metodológicos de la investigación cualitativa); (d) metodologías de enseñanza-aprendizaje. Los resultados destacaron que solo el 40,4% de las disciplinas son obligatorias y hay un predominio de los objetivos de enseñanza-aprendizaje, con foco en la instrumentalización y el estímulo a los estudiantes de posgrado en la elaboración de proyectos de investigación. El 59,3% no informó el tipo de metodología de enseñanza-aprendizaje. Existe una brecha con relación al uso de tecnologías y medios digitales, y el predominio del formato textual. Se encontró debilidad en la formación epistemológica-teórica. Hay un predominio en el aprendizaje del paradigma positivista en relación con el paradigma interpretativo de las teorías/tradiciones críticas. Por último, los elementos se sistematizan en un itinerario formativo con grados crecientes de complejidad, intencionalmente estructurado y desarrollado en un entorno institucional que proporciona decolonialidad y reparación epistémica.

## Introdução

A formação do(a) pesquisador(a) é um fator fundamental para o desenvolvimento e legitimidade da pesquisa qualitativa em saúde. Bosi ^1^ (p. S32) alerta sobre o quanto é “*absolutamente impressionante a lacuna de discussões sobre essa problemática* [da formação] *na literatura científica e em documentos que circulam no meio acadêmico*”.

A que nos referimos como pesquisa qualitativa? Àquela que reúne experiências, percepções e comportamentos dos participantes, buscando responder o como e os porquês ao invés de quantificar ou mensurar os fenômenos, incluindo aqueles que não se submetem à quantificação, caso das produções subjetivas. Nesse sentido, envolve diferentes arranjos entre os polos epistemológico, teórico, axiológico e técnico das ciências sociais, sendo, portanto, plural ao tempo em que se coloca em disputa com outros arranjos e perspectivas científicas. Para tal, segundo Bosi ^2^ (p. 109), é a pesquisa científica que tem “*como material a linguagem e processos de subjetivação em suas várias formas de expressão - ou seja, material qualitativo*”. Ainda, comumente, “*é o reconhecimento de que mais do que explicar, verificar ou predizer, o objetivo deste tipo de pesquisa é compreender e/ou transformar a realidade*” ^3^ (p. 38). Compreender, para Minayo ^4^ (p. 23), “...*é o verbo da pesquisa qualitativa. Compreender relações, valores, atitudes, crenças, hábitos e representações e, a partir desse conjunto de fenômenos humanos gerados socialmente, interpretar a realidade*”.

Ao iniciar a leitura deste artigo, fazemos um convite para que o leitor se questione ou revisite seu percurso em pesquisa ou trajetória na formação em pesquisa qualitativa em saúde. Como aconteceu de se aproximar ou se interessar pelo enfoque qualitativo? Identifica uma leitura, professor(a), problema dos serviços e/ou questão de pesquisa que o demandou? Ao final, após esse olhar retrospectivo, consegue visualizar se em sua trajetória na pesquisa vivenciou um processo de ensino-aprendizagem estruturado institucionalmente, ou de momentos e movimentos de buscas individuais e/ou oportunidades pontuais predominantemente focados na investigação quantitativa? Foi mediada por professores(as) ou grupos de pesquisa para a formação epistemológico-metodológica em pesquisa qualitativa em saúde?

Deslandes & Iriart ^5^ sinalizam que a fragilidade do embasamento teórico-metodológico nos estudos do campo da ciências sociais e humanas em saúde “*sugere um empirismo ateórico*” ^6^ (p. 880). A produção científica de pesquisa qualitativa geralmente não apresenta fundamentação teórica e quando a desenvolve, foca na definição de pesquisa qualitativa utilizada no estudo ou na apresentação “*de algumas de suas tradições particulares, como a fenomenologia ou o construcionismo*” ^7^ (p. 245). Ademais, a ausência de teoria social nas graduações em saúde e o “efeito Babel” apontado por Bosi ^2^, denota uma formação incipiente também do lado dos formadores, são desafios ainda não suficientemente enfrentados pela comunidade de pesquisadores qualitativos na saúde.

Vários estudos ^1^ ^,^ ^8^ ^,^ ^9^ sobre formação de pesquisadores qualitativos evidenciam a necessidade de superar a “ausência” e/ou incipiência do ensino da teoria social ou bases epistemológicas da investigação.

Para Silva et al. ^9^ (p. 632), outro desdobramento do contexto acima descrito é que o “*percurso de pesquisadores iniciantes está muito mais ligado à expertise de seus orientadores do que propriamente a uma cultura acadêmica de formação estruturada em pesquisa qualitativa na área da saúde*”. Eakin ^10^ adensa esse debate ao destacar que a formação em pesquisa qualitativa em saúde não se restringe aos aspectos pedagógicos. A autora desenvolveu uma crítica importante concernente aos contextos ideológicos e institucionais das universidades e institutos de pesquisa no campo da saúde. Ela defende que um “*conteúdo curricular estratégico e uma forte comunidade prática são necessários para dar suporte ao ensino, aprendizado e prática de pesquisa que vai ‘contra a corrente’ do discurso científico dominante e da lógica institucional*” ^10^ (p. 293). Estudiosos(as) da área que se propuseram analisar o ensino da pesquisa qualitativa em saúde na pós-graduação no Brasil, a partir da perspectiva de atores envolvidos, docentes e estudantes afirmam que:

“*A falta de oportunidades de formação na pesquisa qualitativa em saúde no Brasil expressa um dos efeitos da hegemonia do modelo da biomedicina* (...) *Uma rápida consulta às estruturas curriculares dos programas de pós-graduação do campo Saúde Coletiva (considerado como aquele que mais articula o biológico e o social na saúde), realizada a título de exploração não sistemática no decurso desta análise, confirma a assertiva de Bosi* (...) *de que ‘o ensino do enfoque qualitativo ainda se mostra frágil e ocasional, o que* (...) *leva a interrogar se em um campo assumidamente interdisciplinar (trans?) tal pluralidade epistêmica se expressa na formação*’” ^11^ (p. 165).

O reconhecimento de que não há neutralidade no fazer ciência remete, epistemologicamente, ao paradigma interpretativo, quando esse reconhece a reflexividade e a presença do(a) pesquisador(a) Para Bosi ^2^ (p. 126), “*toda pesquisa qualitativa, de um modo ou outro, se inscreve no paradigma interpretativo*”. Ainda é necessário reconhecer que um largo espectro de teorias/tradições teórico-metodológicas se filia a esse paradigma ^12^ ^,^ ^13^ ^,^ ^14^ ^,^ ^15^. A diversidade de pontos de partida ontológicos e metodológicos dessas teorias/tradições e abordagens podem variar de compreensivas a críticas ^1^. Sem promulgar ou defender um único referencial ou teoria/tradição crítica, é apresentado a seguir o que esta investigação assume como lente as teorias sociais críticas.

Ao falarmos em teorias críticas, não remetemos especificamente ao que ficou conhecido como Teoria Crítica da Escola de Frankfurt ^16^. Teorias ou tradições sociais críticas são aquelas que explicitam uma “*postura de pesquisa que problematiza o conhecimento adquirido*” ^16^ (p. 33). É o referencial que manifesta compromisso com desvelar e intervir em situações de injustiças ou iniquidades e, também, alertar para a concentração e as relações de poder. Com essa perspectiva, são sempre interpretativas, utilizando análises não apenas descrições da realidade e sempre questionam as estruturas hegemônicas das instituições estabelecidas, tais como ciência, política, religião, cultura etc. Assim, esse referencial é problematizado, inclusive, mediante a formulação de questões referentes aos tipos de saberes que são reconhecidos ou “não validados” ou desrespeitados no processo de produção e disseminação da pesquisa. Tyson ^17^, no seu capítulo com o curioso título de *Everything You Wanted to Know about Critical Theory, but were Afraid to Ask* (*Tudo o que Você Queria Saber sobre Teoria Crítica, mas Tinha Medo de Perguntar*), aborda alguns dos aspectos acima sistematizados.

Diversos campos do conhecimento, incluindo o da Saúde Coletiva, debatem sobre o epistemicídio de vários saberes pela comunidade científica hegemônica ^17^ ^,^ ^18^ ^,^ ^19^. Maia ^18^ (p. 160) problematiza o processo de “*universalização de uma epistemologia europeia abstrata e universalizante, que subsumiu outras práticas de cognição do mundo*”. O colonialismo, o capitalismo e o patriarcado são analisados de forma articulada na condição de formas de dominação triplas e interseccionais. A expressão dessas “dominações” demanda que pesquisadores(as) resgatem e se aproximem de saberes de “resistência e luta”. Segundo Porto & Martins ^20^ (p. 4), “*implica uma transição paradigmática a partir da valorização de sujeitos, saberes e alternativas de sociedade atualmente oprimidos e invisibilizados*”. Para Marques ^21^ (p. 820): “*É nesse ponto que se insere a convicção antropófaga de que o Brasil tem algo a oferecer ao mundo moderno. O mal-estar brasileiro instala-se no corpo e incita um grito de diálogo com o mundo moderno, acenando para uma linha de fuga do intruso. Se o antropófago podia ignorar o mal-estar, mas não tinha meios para realizar a utopia tecnológica de um século atrás, com a ajuda do STS* [Estudos de Ciência e Tecnologia] *hoje ele tem outra chance (sem garantia de sucesso) de ressurgir, mobilizando-se e articulando-se para mais ciências e conhecimentos inclusivos, menos destrutivos e mais simétricos e democráticos*”.

Agrega-se a esse cenário de crise epistemológico-teórica, o contexto de que, após tantos anos de luta e investimento para institucionalizar a pós-graduação e pesquisa no Brasil, foi implementado nos últimos anos uma política genocida e de desmonte da educação, ciência e tecnologia no país.

Finalmente, a inexistência de um mapeamento sistemático sobre o ensino da pesquisa qualitativa em saúde nos programas de pós-graduação em Saúde Coletiva (PPGSC) no Brasil e o cenário até aqui apresentado originaram as perguntas de pesquisa: como se apresenta o ensino da pesquisa qualitativa dos PPGSC, no Brasil? As disciplinas oportunizam referenciais teóricos, tradições e/ou teórico-metodológicos críticos que favoreçam pluralidade epistemológica e interdisciplinar? Propõe-se então analisar as evidências sobre a formação em pesquisa qualitativa dos PPGSC credenciados pela Coordenação de Aperfeiçoamento de Pessoal de Nível Superior (CAPES) no Brasil no ano de 2021.

Ante o exposto, o foco deste artigo é apresentar e problematizar o lugar da pesquisa qualitativa em saúde nas pós em Saúde Coletiva no Brasil em termos da formação teórico-metodológica e, mais que isso, se essa favorece os(as) pós-graduandos(as) a desenvolverem estudos com teorias(lentes) críticas e propósitos de transformar a realidade com a perspectiva de promoção da equidade e redução das disparidades sociais ^22^ ^,^ ^23^.

## Percurso metodológico

Foi realizado um mapeamento das disciplinas relacionadas à pesquisa qualitativa em todos os PPGSC no Brasil, de setembro a dezembro de 2021, às vésperas da avaliação quadrienal da CAPES, momento em que a maioria dos programas se reorganizam. Para a realização desse mapeamento, foram percorridos os seguintes passos: (i) consulta à Plataforma Sucupira para a localização dos PPGSC em setembro de 2021 (Tabela 1); e (ii) busca nos portais institucionais dos PPGSC localizados para obtenção de informações sobre pesquisa qualitativa de setembro a dezembro de 2021.

**Tabela 1:** Distribuição dos programas de pós-graduação em Saúde Coletiva (PPGSC) segundo região do Brasil e tipo de curso.

Região/Unidade da Federação	Total de PPGSC	Total de cursos	Mestrado	Doutorado	Mestrado Profissional	Doutorado Profissional
Brasil	97	136	52	39	42	3
Região Norte						
Acre	1	2	1	1	-	-
Amazonas	3	3	2	1	-	-
Pará	2	2	2	-	-	-
Região Nordeste						
Bahia	7	10	4	2	4	-
Ceará	5	8	4	3	1	-
Maranhão	2	3	1	1	1	-
Paraíba	2	2	2	-	-	-
Pernambuco	4	6	2	1	2	1
Piauí	1	1	1	-	-	-
Rio Grande do Norte	4	5	2	1	2	-
Sergipe	1	1	-	-	1	-
Região Centro-oeste						
Distrito Federal	3	4	1	1	2	-
Goiás	1	-	-	-	1	-
Mato Grosso do Sul	1	-	-	-	1	-
Mato Grosso	1	2	1	1	-	-
Região Sudeste						
Espírito Santo	1	2	1	1	-	-
Minas Gerais	6	8	2	2	4	-
Rio de Janeiro	20	30	9	8	11	2
São Paulo	17	26	9	11	6	-
Região Sul						
Paraná	2	3	2	1	-	-
Santa Catarina	4	4	2	1	1	-
Rio Grande do Sul	8	11	5	3	3	-

Fonte: elaboração própria, adaptado da Plataforma Sucupira (https://sucupira.capes.gov.br/sucupira/, 2021).

É possível questionar a decisão de consultar páginas dos programas como estratégia para a produção de dados, já que, geralmente, elas não têm como objetivo ser um repositório de ementas e programas detalhados dos componentes curriculares dos cursos. No entanto, especialmente a partir da pandemia, os *sites*, além de serem quesito de visibilidade avaliado na descrição qualitativa da Avaliação Quadrienal de 2017-2020 da CAPES, passaram a ser fontes de informações relevantes aos inscritos nos processos seletivos, aos discentes e aos docentes, bem como ser canal de relacionamento com notícias inerentes ao campo da Saúde Coletiva, e acesso ao Sistema Moodle, à Biblioteca Virtual em Saúde (BVS), CAPES, Conselho Nacional de Desenvolvimento Científico e Tecnológico (CNPq) e outros de interesse da comunidade acadêmica. Também, cabe referir a inexistência dessa informação em outras possíveis fontes como a Plataforma Sucupira, que em períodos anteriores estavam disponíveis nos *Cadernos de Indicadores* do banco de dados da CAPES ^24^.

No entanto, ao acessar os *sites* dos programas de pós-graduação, constatamos a incompletude de informações e, em muitos casos, a não disponibilização de informações acerca das disciplinas ofertadas por eles. Dessa forma, foram definidos os seguintes critérios de inclusão e exclusão das disciplinas: (i) critérios de inclusão: disciplinas que contivessem ementa e bibliografia relacionadas à metodologia da pesquisa; e (ii) critérios de exclusão: disciplinas que, embora abordassem metodologia da pesquisa, estabeleciam o foco nas pesquisas quantitativas ou que informassem, no item bibliografia, que essa seria livre a depender da escolha do(a) professor(a) responsável.

Ao final, foram identificadas 98 disciplinas dos PPGSC que atenderam aos critérios de inclusão conforme sistematizado na Tabela 2.

**Tabela 2:** Relação das instituições de Ensino Superior e cursos com o quantitativo de disciplinas selecionadas para análise segundo região do Brasil, 2021.

Região/Instituição	Programa de pós-graduação	Curso	Disciplinas	Subtotal
			n	n (%)
Norte				4 (3,1)
FIOCRUZ Amazonas	Condições de Vida e Situações de Saúde na Amazônia	Mestrado Profissional	4	
Centro-oeste				7 (7,1)
UFMT	Saúde Coletiva	Mestrado	2	
FIOCRUZ Brasília	Saúde Coletiva	Mestrado	5	
Nordeste				17 (18,4)
FIOCRUZ Pernambuco	Saúde Coletiva	Mestrado, Mestrado Profissional, Doutorado, Doutorado Profissional	4	
UEPB	Saúde Coletiva	Mestrado	1	
UFC	Saúde Coletiva	Mestrado e Doutorado	6	
UFMA	Saúde Coletiva	Mestrado e Doutorado	2	
UFPE	Saúde Coletiva	Mestrado	1	
UFRB	Saúde Negra e Indígena	Mestrado Profissional	3	
Sudeste				56 (57,1)
FIOCRUZ ENSP/UERJ/UFF	Bioética, Ética Aplicada e Saúde Coletiva	Mestrado e Doutorado	2	
FIOCRUZ ENSP	Saúde Pública	Mestrado e Doutorado	15	
FIOCRUZ ENSP	Saúde Pública	Mestrado Profissional	10	
FIOCRUZ IFF	Saúde da Criança e da Mulher	Mestrado e Doutorado	4	
FIOCRUZ Minas Gerais	Saúde Coletiva	Mestrado e Doutorado	2	
UFES	Saúde Coletiva	Mestrado e Doutorado	3	
USP Ribeirão Preto	Saúde Pública	Mestrado e Doutorado	1	
UFRJ	Saúde Coletiva	Mestrado e Doutorado	3	
SES São Paulo	Saúde Coletiva	Mestrado Profissional	1	
UNESA	Saúde da Família	Mestrado e Doutorado	6	
UNESP Botucatu	Saúde Coletiva	Mestrado e Doutorado	2	
UNIFESP	Saúde Coletiva	Mestrado e Doutorado	6	
USP São Paulo	Saúde Coletiva	Mestrado e Doutorado	1	
Sul				14 (14,3)
UNISINOS	Saúde Coletiva	Mestrado	2	
UFRGS	Ensino em Saúde	Mestrado Profissional	4	
UFRGS	Epidemiologia	Mestrado e Doutorado	3	
UFSC	Saúde Coletiva	Mestrado e Doutorado	5	
Total	-	-	-	98 (100,0)

ENSP: Escola Nacional de Saúde Pública Sergio Arouca; FIOCRUZ: Fundação Oswaldo Cruz; IFF: Instituto Nacional de Saúde da Mulher, da Criança e do Adolescente Fernandes Figueira; SES: Secretaria de Estado de Saúde; UEPB: Universidade Estadual da Paraíba; UERJ: Universidade do Estado do Rio de Janeiro; UFC: Universidade Federal do Ceará; UFES: Universidade Federal do Espírito Santo; UFF: Universidade Federal Fluminense; UFMA: Universidade Federal do Maranhão; UFMT: Universidade Federal do Mato Grosso; UFPE: Universidade Federal de Pernambuco; UFRB: Universidade Federal do Recôncavo da Bahia; UFRGS: Universidade Federal do Rio Grande do Sul; UFRJ: Universidade Federal do Rio de Janeiro; UFSC: Universidade Federal de Santa Catarina; UNESA: Universidade Estácio de Sá; UNESP: Universidade Estadual Paulista;

UNIFESP: Universidade Federal de São Paulo; UNISINOS: Universidade do Vale do Rio dos Sinos; USP: Universidade de São Paulo.

A partir desse universo de disciplinas, foram lidas todas as ementas disponíveis e a análise foi realizada de forma independente por três das autoras. Para favorecer a “homogeneidade/padronização” da categorização, as pesquisadoras iniciaram analisando conjuntamente uma das regiões com menor número de disciplinas. Os dados foram registrados em planilha de Excel Office 365 (https://products.office.com/). Utilizando uma análise temática reflexiva ^25^ ^,^ ^26^ com movimentos indutivos e dedutivos, foi possível agrupar a produção dos dados e caracterização das disciplinas (código D1-D110) nas seguintes macrocategorias de análise: carga horária e modalidade de oferta (obrigatória ou eletiva); objetivos de ensino-aprendizagens; conteúdos (com destaque para investigação dos paradigmas científicos ou diferentes abordagens teórico-metodológicas da investigação qualitativa); metodologias de ensino-aprendizagens. Ainda foram identificados nomes das disciplinas, referências e autores.

Os objetivos de ensino-aprendizagem foram subdivididos em: (i) instrumentalizar - foco na elaboração e acompanhamento dos projetos dos(as) pós-graduandos(as); (ii) valorizar/sensibilizar pesquisas qualitativas - investe no atitudinal; reflexividade; relação pesquisador(a)-pesquisado(a) (aplicabilidade entendida como vantagens/indicações da pesquisa qualitativa e produtos) e (iii) fundamentar - predomina noções gerais; princípios e/ou conceitos fundamentais da pesquisa qualitativa ou “ferramentas conceituais” ^27^. Essa classificação se fundamenta em uma sistematização de Anastasiou ^27^, que os diferencia dos tipos de aprendizagem e características de conteúdos procedimentais, atitudinais e de conceituais.

Quanto aos conteúdos, foram agrupados em: (1) positivista; (2) interpretativo segundo referencial de Bosi ^2^ e, ainda nesse segundo paradigma, indicamos um terceiro (3), no qual a interpretação é guiada pelas teorias/tradição críticas, ou seja, nos quais identificamos teorias/tradições do paradigma interpretativo que são críticas. Distinguimos que (2) diz respeito ao paradigma interpretativo circunscrito às tradições/teorias do tipo compreensiva. Já (3) se refere também ao paradigma interpretativo, mas com recorte das disciplinas que evidenciaram o ensino fundamentado nas teorias e tradições críticas ^16^.

O plano de análise foi estruturado em três eixos ^1^ ^,^ ^28^ ^,^ ^29^ orientados pelas seguintes questões:

(a) Eixo ontológico: qual a natureza da realidade e do mundo? Quais compreensões de sociedade, saúde, justiça-injustiça têm sido privilegiadas na formação da pesquisa qualitativa?

(b) Eixo epistemológico: como estamos construindo o conhecimento? Qual relação e/ou tipos de relação entre investigador e investigado? Quais saberes têm sido priorizados e veiculados? Quais os papéis e as compreensões de pesquisa científica têm predominantemente orientado a pesquisa qualitativa dos nossos programas?

(c) Eixo praxiológico: como temos atuado e como propomos transformar? Quais horizontes de transformações e sentidos?

## Resultados e discussão

Após a aplicação dos critérios de inclusão e exclusão, foram eliminadas, conforme já aludido, disciplinas com foco na metodologia da pesquisa quantitativa e bibliografia livre.

### Mapeamento das disciplinas segundo carga horária e oferta nos programas

Na Tabela 2, foi evidenciado que a Região Sudeste ficou com mais de 50% das disciplinas que foram incluídas no estudo. Esse fato provavelmente se associa à maior quantidade de programas nessa região (57,1%), cuja análise tem sido aprofundada por diversos estudos ^28^ ^,^ ^30^ ^,^ ^31^.

A Tabela 3 identifica a modalidade de oferta (obrigatória ou eletiva) de 52 disciplinas, correspondendo a 53,1% das 98 mapeadas nos programas de todas as regiões. Nas demais, não foi possível verificar a partir dos *sites*.

**Tabela 3:** Distribuição das disciplinas segundo tipo de oferta e região do Brasil, 2021.

Região	Tipo de disciplina	
	Obrigatória	Eletiva
	n (%)	n (%)
Centro-oeste	5 (9,6)	1 (1,9)
Norte	0 (0,0)	1 (1,9)
Nordeste	6 (11,5)	3 (5,8)
Sudeste	4 (7,7)	19 (36,5)
Sul	6 (11,5)	7 (13,5)
Total	21 (40,4)	31 (59,6)

A escassez da oferta de disciplinas específicas e obrigatórias de pesquisa qualitativa em saúde nos PPGSC, desdobra o que já se observa nas graduações no campo. É possível afirmar que a situação é agravada pela não definição de um percurso formativo distinto entre o mestrado e o doutorado ^11^ ^,^ ^32^. Uma das consequências tem sido a aprendizagem centrada em técnicas/procedimentos e com poucas oportunidades de formação teórico-metodológica.

Esse resultado converge com o estudo de Bosi ^1^, que evidenciou “formação ocasional”, e com o de Conceição ^11^, que mostra que, nas instituições que oferecem disciplina de pesquisa qualitativa em saúde, elas são de “natureza eletiva”, refletindo “*um indicativo da menoridade acadêmica da abordagem qualitativa*” ^11^ (p. 27) nos programas, reflexo da posição desse enfoque no campo da saúde. A acumulação de capital científico (e outros tipos), tende a retroalimentar grupos já consolidados e contrariar, no espaço acadêmico, a equidade defendida pelo movimento da Reforma Sanitária Brasileira.

“*Nas comunidades acadêmicas em que tal visão prospera, conforme acredito ser o caso da Saúde Coletiva brasileira, verifica-se um abandono gradual da dimensão teleológica inerente à produção de conhecimento, reduzindo a avaliação científica a contabilidades mecânicas. Mais que isso, tal processo se beneficia, ao tempo em que reifica, da fetichização de veículos internacionais, já que se constrói a crença na desqualificação da produção e dos veículos nacionais, que bem sabemos não ser justa*” ^33^ (p. 2389).

Vale ainda mencionar que essa lacuna (de formação em pesquisa qualitativa) é uma das expressões da distribuição desigual do capital científico no campo, com desdobramentos nos campos simbólico, político e econômico, uma vez que define a “boa forma de fazer ciência”, os espaços ocupados nas revistas, além das seleções em editais e ocupação de lugares de prestígio ^24^.

#### Objetivos de aprendizagem priorizados nas disciplinas

A definição dos objetivos de ensino-aprendizagem comumente orienta os conteúdos e a seleção de estratégias educativas. No estudo em tela, os objetivos de aprendizagem predominantemente identificados nas disciplinas visavam instrumentalizar e/ou habilitar os(as) pós-graduandos(as) na construção dos projetos de pesquisa. Esse foco na instrumentalização se refletiu na quantidade de referências bibliográficas direcionadas para essa finalidade. Essas foram categorizadas a partir das etapas de desenvolvimento da pesquisa: planejamento, produção ou análise de “dados”. A Tabela 4 evidencia que a maioria das disciplinas contemplou os três tipos de objetivos educacionais, todas identificadas com orientação do paradigma interpretativo com teoria/tradição crítica, e que investiram no objetivo de valorizar e sensibilizar, priorizando a formação de valores e/ou atitudinal.

**Tabela 4:** Distribuição das disciplinas segundo paradigmas e objetivos educacionais por região.

Paradigma/Objetivos	Região					Total
	Centro-oeste	Norte	Nordeste	Sudeste	Sul	
Positivista	4	0	0	23	2	29
Instrumentalizar	4	0	0	22	2	28
Valorizar/Sensibilizar	0	0	0	17	1	18
Fundamentar	0	0	0	21	1	22
Interpretativo	3	2	12	24	12	53
Instrumentalizar	3	2	11	24	11	51
Valorizar/Sensibilizar	2	1	11	23	11	48
Fundamentar	2	2	8	24	7	43
Interpretativo/Tradição crítica	0	2	5	9	0	16
Instrumentalizar	0	2	4	9	0	15
Valorizar/Sensibilizar	0	2	5	9	0	16
Fundamentar	0	2	0	9	0	11

Observou-se uma recorrência de referências relacionadas a abordagens metodológicas, destacando a prevalência para estudos de cunho etnográficos, além de cartografia e observação participante. Entre as técnicas de produção de “dados”, a entrevista foi a mais citada. Para os procedimentos de análise e interpretação, análise do discurso e análise de conteúdo foram os mais listados. Foi possível identificar as tendências metodológicas e a hegemonia de certos modos de investigar a realidade, produzir material e interpretá-los.

Essa compilação de métodos reflete e dialoga com as observações de Deslandes & Maksud ^24^ (p. 12): “*se o domínio científico disciplinar é reconhecido como uma condição para o bom exercício da reflexão interdisciplinar, a formação de maior natureza operativa e instrumental pode minar o projeto de reflexão guiado pela perspectiva da complexidade, característico do ideário da Saúde Coletiva*”.

Percebeu-se, ainda, uma lacuna quanto à utilização das tecnologias e ambientes digitais para a pesquisa em saúde. Há um predomínio absoluto do formato textual e total ausência do modal (janelas pop-up, modais User Experience (UX) ou janelas pop-up modais que utilizam Hypertext Markup Language (HTML), Cascading Style Sheets (CSS), JavaScript e outras) e do imagético (foto, voz, desenhos, modelagem e outros). Com a ampliação dos ambientes digitais, há necessidade de compreender novas epistemologias e metodologias para compreensão das sociabilidades decorrentes dos novos dispositivos da vida cotidiana, considerando que “*a socialidade assumirá novos contornos a partir do marco tecnológico definido como Internet 2.0*” ^34^ (p. 2). A ruptura de fronteiras entre real/virtual e a pesquisa mediada por tecnologias, já é uma realidade que acena para novos desafios que envolvem o trabalho de campo, no qual as fronteiras deixam de ser territoriais e passam a ser simbólicas, a produção discursiva passa a ser caracterizada pelo caráter efêmero e volátil do meio digital e, ainda, pelas diversas possibilidades a ele inerentes.

#### Formação epistemológico-metodológica e teorias/tradições priorizadas nas disciplinas

Quanto ao tipo de paradigma científico ou referencial teórico-metodológico ensinado/apresentado nos programas, 16 disciplinas (16,3%) foram categorizadas como de conteúdo do tipo interpretativo-tradição crítica.

Constatou-se a fragilidade na formação epistemológico-teórica, especialmente relacionada às teorias críticas. Esse resultado não surpreendeu, pois para Bosi & Gastaldo ^35^ (p. 26), há uma “*inexistência quase absoluta de literatura voltada ao tema dos fundamentos teórico-metodológicos no ensino da pesquisa qualitativa no âmbito da saúde (coletiva)*”. Ainda, segundo Bosi ^1^ (p. S34), “*o modelo formador nega a subjetividade como objeto e neutraliza a subjetividade do educando*”.

O predomínio de formação fundamentada no paradigma interpretativo com ênfase na teoria da complexidade difere dos achados do estudo de Russo & Carrara ^36^. Esses autores ao analisarem historicamente o percurso das Ciências Sociais no campo da Saúde Coletiva, a partir de trabalhos publicados sobre o tema, constataram que predominava “*corte marxista e ciência política*” ^36^ (p. 475) seguida das “*perspectivas socioantropológicas, em aportes compreensivistas e construtivistas*” ^24^ (p. 3). A influência do pensamento eurocêntrico, presente na formação do pensamento social brasileiro ^19^ ^,^ ^29^ ^,^ ^37^, também se manifestou neste estudo. Embora seja considerado que a Saúde Coletiva está situada como um novo movimento com raízes históricas no campo acadêmico progressista, ainda é notável a necessidade de expansão das lentes de dominação epistêmicas resultantes do imperialismo, do colonialismo e do patriarcado.

Entre as teorias/tradições críticas predominaram abordagens de pesquisas participativas com diferentes denominações: pesquisa participativa (3); pesquisa ação (3); pesquisa ação participativa (3); pesquisa participativa baseada na comunidade (3), incluindo uma com referência à comunidade ampliada de pesquisa. Wallerstein ^37^ realiza uma importante análise da trajetória de desenvolvimento e produção científica das teorias/tradições que fundamentam as abordagens das pesquisas participativas. Diversas denominações são utilizadas quando se pretende a conexão da ação social contra as iniquidades em saúde.

No contexto brasileiro, podemos destacar, como exemplos, a pesquisa-ação protagonizada por Michel Thiollent ^38^, que se pauta na incorporação de uma multiplicidade de atores comprometidos com a mudança social. A pesquisa-intervenção, modalidade também expressiva no âmbito da saúde, se orienta a partir da cocriação de um “*processo de pesquisa que provoca transformações e mobiliza forças no campo investigado, incluindo-se nele o próprio pesquisador*” ^39^ (p. 143). E a pesquisa participante em saúde que se orienta pelos valores de Paulo Freire, sendo esses sujeitos ativos e participativos e não objetos de estudo.

Nas considerações de Wallerstein ^37^, as especificidades do termo utilizado não são tão importantes, embora reconheça que a nomenclatura utilizada pode colaborar com o avanço dessas teorias, mas que o compromisso está em “*não fazer pesquisa ‘sobre’ uma comunidade e não fazer pesquisa ‘em’ um ambiente comunitário, mas fazer pesquisas ‘com’ parceiros da comunidade*” ^38^ (p. 15). Para tanto, a colaboração entre as partes multissetoriais interessadas deve estar comprometida com o desenvolvimento de prioridades da comunidade para a criação de estratégias que promovam a saúde e a equidade.

A partir da análise das teorias/tradições das disciplinas com formação em teorias críticas, evidenciou-se ainda, uma lacuna ou incipiência de referenciais críticos como “feminismos”, “pensamento negro” e decolonialidade. Enquanto a decolonialidade integrou o conteúdo de cinco disciplinas, epistemologias do Sul foi referenciada somente em uma. Essas teorias se apresentaram com maior presença em cursos criados mais recentemente, cujas temáticas ou nomeação dos programas já indicam um posicionamento de diálogo de saberes. Esses cursos explicitaram objetivos educacionais e conteúdo com intencionalidade de descolonizar poder, saber e pensar. Assim, suas disciplinas se alinham com propostas interseccionais e decoloniais na medida em que se propõem a entender as relações sociais, marcadas pela diversidade das experiências cotidianas. Portanto, consideram categorias como raça, etnia, nacionalidade, orientação sexual, gênero, entre outras que se interrelacionam, de modo a abranger sua complexidade ^40^.

Santos ^41^ (p. 28) aponta para a ecologia dos saberes entendendo “*o reconhecimento da copresença de diferentes saberes e a necessidade de estudar as afinidades, as divergências, as complementaridades e as contradições que existem entre eles, a fim de maximizar a eficácia das lutas de resistência contra a opressão*” a partir de uma perspectiva contra-hegemônica à superação da utilização exclusiva dos saberes eurocêntricos.

Esse diálogo de saberes, além de favorecer projetos com a população e /ou grupos populacionais em contexto de maior iniquidade, tem potencial de incidir sobre as produções do programa que atendam ao fator de impacto da última avaliação quadrienal da CAPES (2017-2020) ^42^: “*evidências de articulação na sua produção ou circulação com a sociedade civil e movimentos sociais, assim como para subsidiar políticas públicas de saúde ou intersetoriais*”.

Ao investigar a interdisciplinaridade dos conteúdos e referenciais explicitados nas disciplinas, foi possível identificar o diálogo ou menção às seguintes disciplinas: filosofia da ciência; antropologia; sociologia da ciência; literatura; história; estética e arte. Houve predomínio da filosofia da ciência, no entanto, com escassez de dialogicidades epistemológicas.

#### Quanto às metodologias de ensino-aprendizagem

A maioria das disciplinas não apresentou dados sobre a metodologia utilizada no processo ensino-aprendizagem na formação em pesquisa qualitativa em saúde. Das 54 disciplinas classificadas como interpretativas, 59,3% não informaram o tipo de metodologia. Entre as que explicitaram, 27,8% focaram nos projetos de pesquisa de estudantes. O domínio do referencial teórico também foi detalhado e/ou listado no tópico das disciplinas referente às metodologias utilizadas (14,8%). Outro investimento das disciplinas diz respeito à instrumentalização para a análise de dados (7%), incluindo demonstração de softwares. Houve ainda uma pequena parte voltada para a instrumentalização, visando a produção dos dados, a escrita de artigos e debates de textos e vídeos (3,7% para cada).

Destacamos que a exposição dialogada e/ou apresentação dos(as) docentes e a realização de seminários pelos(as) estudantes foram as estratégias pedagógicas mais mencionadas pelas disciplinas categorizadas como positivistas e interpretativas. A distinção entre elas é que, nas interpretativas, predominaram seminários de estudantes e outras estratégias de metodologias ativas de aprendizagem. Entre as atividades práticas explicitadas chamou atenção a: “*análise crítica sobre um artigo científico publicado de abordagem qualitativa, avaliando a consistência de todos os itens em seus conteúdos e forma, bem como a coerência entre objetivos, método empregado, técnicas/instrumentos de coleta de informações aplicados, análise dos dados e apresentação dos aspectos éticos*” (D57). Uma vez mais, registra-se o silenciamento ou ausência da fundamentação teórica e/ou epistemológico-teórica como critério de análise e/ou de qualidade do rigor metodológico da pesquisa qualitativa em saúde.

Essa lacuna requer o resgate de um dos pressupostos já apresentados na introdução deste artigo: “*ensinar pesquisa qualitativa em saúde requer um entendimento onto-epistemológico alicerçado na filosofia e nas ciências sociais*” ^43^ (p. 592). Das 16 (16,3%) disciplinas que foram classificadas como interpretativa/crítica, metade não menciona a metodologia utilizada. Entre aquelas que foram possíveis identificar, todas tiveram como foco o projeto dos estudantes e o referencial teórico, com pequenas diferenças nas dinâmicas, visando oportunizar aplicação ou prática em ambiente pedagógico. Algumas descrevem estratégias que incluem o(a) docente, a exemplo do “*debate-formativo para o aluno e seu orientador*”. Entre as estratégias pedagógicas com potencial inovador podem ser citadas: “*Vivência da teoria e os procedimentos da análise de discurso - ler, descrever e interpretar - em suas variadas formas de manifestação: oral, texto, figura, imagem. Discutir os elementos que compõem o discurso com ênfase na memória, no gesto, no verbal, no não-verbal e no silêncio*”. “*Construção de conceitos e categorias de análise*” (D7, D74). “*Exercícios de trabalho de campo, supervisionados pelo responsável da disciplina, através dos quais os alunos poderão ter contato com alguns procedimentos de pesquisa, como observação e visita de campo*” (D7, D75, D79, D94).

Conceição et al. ^11^, em um estudo sobre educação de pesquisadores qualitativos, segundo perspectiva de discentes e docentes, identificaram que uma das estratégias para inovar nessa formação seria a “*adoção de atividades mais centradas no processo de aprendizagem dos estudantes do que no ensino de conteúdos estanques*” ^11^ (p. 17). Para tal, sugerem “*a construção de uma rede de suporte didático-pedagógico nacional e internacional para que os docentes possam compartilhar experiências e estratégias didático-pedagógicas*” ^11^ (p. 18).

Finalmente, respondendo às questões apresentadas nos eixos do plano de análise da metodologia da pesquisa, este estudo evidenciou que o paradigma positivista predomina em relação ao paradigma interpretativo de teorias/tradições críticas. Dessa forma, a compreensão da sociedade e da saúde se ancora na busca da objetividade, da verdade e em uma pretensa neutralidade diante da realidade. No eixo epistemológico, tende a uma relação pesquisador(a)-pesquisador(a) com verticalização dos saberes e assimetria de poder. O eixo praxiológico sinaliza que os sentidos ainda estão centrados no(a) investigador(a) e com poucos elementos para uma transformação que considere as perspectivas das pessoas e/ou grupos envolvidos na produção dos dados. Não foram identificadas, na descrição das disciplinas, estratégias de devolução, disponibilização dos resultados e/ou tradução dos conhecimentos produzidos. Uma disciplina sinalizou a estratégia de comunidade ampliada de pesquisa, na qual os(as) participantes coconstroem das perguntas de pesquisa à análise e tradução dos resultados.

## Considerações finais

A partir do reconhecimento que todo estudo reflete a temporalidade e contexto histórico no qual está inserido, admite-se que, com a proximidade e realização da avaliação quadrienal 2017-2020 da CAPES em 2022, possam ter sido atualizados os *sites* e/ou criadas disciplinas nos programas de pós participantes deste estudo. Também com o contexto entre pandemias, muitas disciplinas foram revistas e atualizadas, considerando a ambiência *online* e /ou modalidades híbridas de oferta da pós-graduação brasileira.

Um dos limites do estudo referente à sua construção metodológica foi o pequeno número de disciplinas com completude de informação para ser incluída no *corpus* de análise. Por outro lado, os achados evidenciaram pistas para a reconstrução crítica dos referenciais a serem oferecidos nos programas de Saúde Coletiva.

O ensino centrado na instrumentalização ou na “metodolatria” ^8^ e o predomínio da retórica das evidências e dos critérios de qualidade ainda focam nas métricas da produtividade e dificultam o investimento na reflexividade e fundamentação do(a) pesquisador(a).

O investimento em um quadro teórico denso e congruente que favoreça tanto a legitimidade quanto a relevância da pesquisa qualitativa no campo científico ^2^ ^,^ ^44^, requer um itinerário formativo com graus crescentes de complexidade, intencionalmente estruturado em um ambiente institucional que propicie decolonialidade e reparação epistêmica. O contexto latino-americano e, em especial, o brasileiro, oferecem condições de revigorar a subjugação do conhecimento a novas narrativas.

Quanto às metodologias de ensino-aprendizagem, destaca-se como um potencial de inovação nas disciplinas analisadas, a possibilidade dos(as) estudantes vivenciarem as teorias e os procedimentos da análise de discurso - ler, descrever e interpretar - em suas variadas formas de manifestação: oral, texto, figura, imagem. Também, o exercício de percepção do gesto, do verbal, do não-verbal e do silêncio.

A formação disciplinar Ciências Humanas e Sociais, a despeito de seu lugar na ruptura epistemológica que funda o campo Saúde Coletiva, não encontra espaço equivalente ao paradigma positivista nos PPGSC. Considerando que muitos alunos (ainda que não a maioria) são procedentes de cursos no campo das Ciências Humanas e Sociais - notadamente psicologia, educação, sociologia - e, tal como os alunos do campo da saúde, apresentam dificuldades de compreensão, por vezes, equivalentes, cabe indagar como ocorre a formação nesses domínios. Trata-se de uma questão de pesquisa que ultrapassa o escopo deste artigo, mas importa sobremaneira para a comunidade de pesquisadores qualitativos em sua busca de expansão com rigor.

É imprescindível que, a partir das universidades, do fórum de Pós-graduação em Saúde Coletiva e/ou da Comissão de Ciências Sociais e Humanas em Saúde da Associação Brasileira de Saúde Coletiva (Abrasco), seja feito um esforço conjunto para aumentar a representação e o impacto da pesquisa qualitativa nos currículos e programas das disciplinas da pós-graduação brasileira. O itinerário formativo deve ser intencionalmente estruturado e desenvolvido em um ambiente institucional que propicie decolonialidade e reparação epistêmica.

Investir nas dimensões epistemológico-metodológica, ético-política e não se restringir à dimensão técnico-operacional pode ser um caminho para desvelar e intervir em situações de injustiça e iniquidades, assim como valorizar grupos populacionais e saberes socialmente invisibilizados.
